# *Porphyromonas gingivalis* Induces Proinflammatory Cytokine Expression Leading to Apoptotic Death through the Oxidative Stress/NF-κB Pathway in Brain Endothelial Cells

**DOI:** 10.3390/cells10113033

**Published:** 2021-11-05

**Authors:** Vichuda Charoensaensuk, Yen-Chou Chen, Yun-Ho Lin, Keng-Liang Ou, Liang-Yo Yang, Dah-Yuu Lu

**Affiliations:** 1 School of Dentistry, College of Oral Medicine, Taipei Medical University, Taipei 11031, Taiwan; d825104004@tmu.edu.tw (V.C.); kevinyhl@tmu.edu.tw (Y.-H.L.); 2 Graduate Institute of Medical Sciences, College of Medicine, Taipei Medical University, Taipei 11031, Taiwan; yc3270@tmu.edu.tw; 3 3D Global Biotech Inc., New Taipei City 22175, Taiwan; klouyu@gmail.com; 4 Department of Physiology, School of Medicine, College of Medicine, China Medical University, Taichung 40402, Taiwan; 5 Laboratory for Neural Repair, China Medical University Hospital, Taichung 40447, Taiwan; 6 Department of Pharmacology, School of Medicine, College of Medicine, China Medical University, Taichung 40402, Taiwan; 7 Department of Photonics and Communication Engineering, Asia University, Taichung 41354, Taiwan

**Keywords:** *P. gingivalis*, ROS, NAC, cell apoptosis, proinflammatory cytokine

## Abstract

*Porphyromonas gingivalis*, a periodontal pathogen, has been proposed to cause blood vessel injury leading to cerebrovascular diseases such as stroke. Brain endothelial cells compose the blood-brain barrier that protects homeostasis of the central nervous system. However, whether *P. gingivalis* causes the death of endothelial cells and the underlying mechanisms remain unclear. This study aimed to investigate the impact and regulatory mechanisms of *P. gingivalis* infection in brain endothelial cells. We used bEnd.3 cells and primary mouse endothelial cells to assess the effects of *P. gingivalis* on endothelial cells. Our results showed that infection with live *P. gingivalis*, unlike heat-killed *P. gingivalis*, triggers brain endothelial cell death by inducing cell apoptosis. Moreover, *P. gingivalis* infection increased intracellular reactive oxygen species (ROS) production, activated NF-κB, and up-regulated the expression of IL-1β and TNF-α. Furthermore, *N*-acetyl-L-cysteine (NAC), a most frequently used antioxidant, treatment significantly reduced *P. gingivalis-*induced cell apoptosis and brain endothelial cell death. The enhancement of ROS production, NF-κB p65 activation, and proinflammatory cytokine expression was also attenuated by NAC treatment. The impact of *P. gingivalis* on brain endothelial cells was also confirmed using adult primary mouse brain endothelial cells (MBECs). In summary, our results showed that *P. gingivalis* up-regulates IL-1β and TNF-α protein expression, which consequently causes cell death of brain endothelial cells through the ROS/NF-κB pathway. Our results, together with the results of previous case-control studies and epidemiologic reports, strongly support the hypothesis that periodontal infection increases the risk of developing cerebrovascular disease.

## 1. Introduction

Periodontal disease is a chronic inflammatory disease that affects the gums, disrupts tooth-supporting tissues, exacerbates bone resorption, and causes tooth loss [1]. Advanced forms of periodontitis, including both severe aggressive periodontitis (mainly affects adolescents or young adults) and severe chronic periodontitis (mainly affects adults), affect 10–15% of the population worldwide [2]. After inadequate oral hygiene disrupts the oral microflora balance, bacterial colonization and biofilm production interact with the host’s immune response, resulting in inflammation and disease [3]. If left untreated, periodontal disease may lead to systemic inflammation [2]. The black-pigmented Gram-negative anaerobe, *Porphyromonas gingivalis,* is a primary microorganism that causes periodontal disease. Daily activities, such as tooth brushing, scaling, extraction, and other dental procedures, can allow *P. gingivalis* and its virulence factors to enter the systemic vascular system [4]. Moreover, the molecular signatures of *P. gingivalis* have been discovered in the brain tissues (other than the oral cavity) of patients with Alzheimer’s Disease, which are also associated with systemic proinflammatory state [5]. Endothelial cell damage, dysfunction, and activation are observed during the progression of atherosclerosis [6]. Active and invasive *P. gingivalis* have been detected in human atherosclerotic plaque tissues [7]. Periodontal disease has also been associated with an increased risk of developing rheumatoid arthritis, cardiovascular disease (CVD), and other systemic diseases [8,9]. In fact, *P. gingivalis*, possesses a unique microbial enzyme, peptidylarginine deiminase (PAD), the human equivalent of which has been identified as a susceptibility factor for rheumatoid arthritis [10]. Increasing epidemiologic studies have identified plausible molecular pathways that demonstrate a causative link between periodontal infection and CVD [11,12,13,14]. There is a correlation between periodontal infection and CVD [15,16], and periodontal disease treatment has favorable effects on CVD [17,18].

The biological links and interconnections of the oral-brain axis are an area of growing interest between the oral microbiota and the pathophysiology of brain disorders. In particular, gingipains from *P. gingivalis* have also been found in brain tissues [19]. Live *P. gingivalis* and its virulence factors are powerful intracerebral inflammatory initiators, which have direct implications on memory and lesion development [20]. Several case-control and epidemiologic studies have suggested that clinical periodontitis is associated with a higher risk of stroke [21,22]. Pussinen et al. [23] found an increase in IgA antibodies to *P. gingivalis*, which indicated a recurrent stroke in participants with a history of stroke. According to Ghizoni et al. [24], stroke patients have deeper pockets, more extensive attachment loss, higher plaque indexes, and higher levels of *P. gingivalis* in their pockets. Prospective clinical studies have implicated a relationship between periodontitis and major depression [25]. Recently, *P. gingivalis* gingipains have been considered potential therapeutic targets for sporadic Alzheimer’s disease (AD) [26]. *P. gingivalis* was observed to invade and persist in mature neuronal cells and shows signs of AD-like neuropathology [27]. Surprisingly, *P. gingivalis* has been detected in cerebrospinal fluid and is present in the brains of over 90% of patients with AD [19]. However, with only case-control and epidemiologic reports, the strength of the association between periodontal infection and stroke is still quite weak. Brain endothelial cells align to form a lining membrane that prevents the brain from circulating harmful substances in the bloodstream, suggesting that brain endothelial cells directly contact with pathogens in the bloodstream [28]. Our previous study has reported that the arrangement of brain endothelial cells maintains the structure of the brain-blood barrier (BBB) and tight junction formation and destruction of brain endothelial cells causes hyperpermeability of the BBB [29]. We also found inhibition of brain endothelial cell damage, which protects the BBB from injury caused by cerebral ischemia/reperfusion in an animal model [30].

Emerging studies have shown that intracellular reactive oxygen species (ROS) induces signal transduction that triggers the up-regulation of inflammatory cytokines [31]. Various exogenous factors, including bacterial infection and their virulence factors, stimulate intracellular ROS production [32,33]. Elevated ROS production and oxidative stress in many diseases, such as cancer, CVD, and bacterial infection, cause damage to DNA, proteins, and tissues and further cause inflammation and cellular apoptosis [34,35,36,37]. Periodontitis patients were more sensitive to the apoptotic effect induced by TNF-related apoptosis-inducing ligand (TRAIL), leading in this way, to alveolar bone loss [38,39]. Evidence has shown that ROS contributes to the pathogenesis of several nervous system diseases such as stroke, AD, and Parkinson’s disease [40,41]. We reported that the induction of ROS production in the central nervous system causes neuroinflammation and may lead to neurodegeneration [42]. Moreover, our previous study also found that inhibition of ROS production in the brain improved motor performance in a mouse model [43]. Antioxidants that prevent or reduce the production of ROS have beneficial effects on several neural disorders. *N*-acetyl-L-cysteine (NAC), an acetylated cysteine residue with a free thiol group, interacts with the electrophilic groups of ROS and offer direct and indirect antioxidant properties. Previous studies have shown that NAC exerts beneficial therapeutic effects in several neurological disorders [44,45,46].

Previously, we reported that *Helicobacter pylori* infection induces TLR4 signaling and IL-8 expression in gastric epithelial cells [47]. This study aimed to investigate the underlying mechanism of *P. gingivalis* infection in brain vascular cells. We hypothesized a link between *P. gingivalis* infection in endothelial cells in terms of inflammatory response, cell apoptosis, and brain endothelial cell death. We also used the antioxidant NAC, which scavenged ROS levels, to further confirm oxidative stress, proinflammatory cytokine expression, and cell death caused by *P. gingivalis* infection. This study provides a connection between periodontal infection and cerebrovascular diseases.

## 2. Materials and Methods

### 2.1. Animals

Six to seven-week-old male C57BL/6 mice were obtained from BioLASCO (Taipei, Taiwan) and bred in a temperature- and humidity-controlled environment. Mouse were given access to food and water ad libitum. All animal procedures were approved by Institutional Animal Care and Use Committee, China Medical University (Taichung, Taiwan; CMUIACUC-2020-277) and carried out in accordance with the Animal Care and Use Guidelines of China Medical University (Taichung, Taiwan).

### 2.2. Cell Culture

Mouse brain endothelial cells (bEnd.3; ATCC^®^ CRL-2299™; American Type Culture Collection; ATCC; Manassas, VA, USA) were cultured in Dulbecco’s modified eagle medium (DMEM; Cat. # 12100046; Gibco, Grand Island, NY, USA) supplemented with 10% fetal bovine serum (FBS; Cat. # 26140079; Gibco, Grand Island, NY, USA) and 1% Penicillin Streptomycin (Pen Strep; Cat. # 15140122; Gibco, Grand Island, NY, USA) in a CO_2_ incubator at 37 °C with a humidified atmosphere of 95% air and 5% CO_2_.

### 2.3. Primary Cell Isolation and Culture

The animal study was approved by Institutional Animal Care and Use Committee (IACAC), China Medical University (Taichung, Taiwan; CMUIACUC-2020-277). Primary mouse brain endothelial cells (MBECs) were isolated from 7 to 8-week-old male C57BL/6 mice according to the protocol reported by [48,49]. Typically, 15 mice were used for each isolation. Mice were under anesthesia before decapitated. Brains were excised and stored in DMEM containing 2% Pen Strep on ice. Cerebellum and olfactory bulb were cut off and meninges were rolled out on sterile filter papers. After that, mechanical digestion was carried out by mincing brain tissues and pressing the minced tissues through 18-G and 21-G needles, respectively. The homogenate was digested for 75 min at 37 °C on a shaker incubator at 200 rpm in DMEM with 1.05 mg/mL type II collagenase (Cat. # C6885; Sigma-Aldrich, St. Louis, MO, USA) and 58.5 U/mL type I DNase (Cat. # DN25; Sigma-Aldrich, St. Louis, MO, USA). After stopping the digestion with ice-cold DMEM, the homogenate was collected by centrifugation at 800× *g* for 8 min at 4 °C. To remove myelin sheath, pellet was resuspended with 20% bovine serum albumin (BSA; Cat. # A9647; Sigma-Aldrich, St. Louis, MO, USA) in DMEM and centrifuged (1000× *g*, 20 min, 4 °C). The pellet underwent a second digestion with 1 mg/mL collagenase/dispase (Cat. # 10269638001; Roche, Basel, Switzerland) and 39 U/mL type I DNase for 75 min at 37 °C on a shaker incubator (200 rpm). Then, cellular contaminant was removed from microvessels by the centrifugation with 33% continuous Percoll gradient (Cat. # 17089102; GE Healthcare, Chicago, IL, USA) at 700× *g* for 10 min, 4 °C. Microvessels were resuspended with DMEM and centrifuges at 800× *g* for 8 min, 4 °C to remove Percoll residues prior to the culture. Afterwards, microvessels were cultured on cell culture flasks coated for 4 h with type IV collagen (Cat. # C5533; Sigma-Aldrich, St. Louis, MO, USA) 5 μg/cm^2^. MBECs were maintained in DMEM supplemented with 20% FBS, 1% Pen Strep, 1 ng/mL basic fibroblast growth factor (bFGF; Cat. # ab217391; Abcam, Cambridge, UK), 100 μg/mL sodium heparin (Cat. # H3393; Sigma-Aldrich, St. Louis, MO, USA), 1.4 μM hydrocortisone (Cat. # H0888; Sigma-Aldrich, St. Louis, MO, USA), 0.2% Insulin-Transferrin-Sodium Selenium supplement (ITS; Cat. # I3146; Sigma-Aldrich, St. Louis, MO, USA) in a CO_2_ incubator at 37 °C with a humidified atmosphere of 95% air and 5% CO_2_. Puromycin 4 μg/mL (Cat. # P8833; Sigma-Aldrich, St. Louis, MO, USA) was added to the cell culture medium at the first two days of culture to purify the cells before switching back to regular culture medium for MBECs. Cells were passaged using TrypLE™ express enzyme (Cat. # 12604021; Gibco, Grand Island, NY, USA) once they achieved 90–95% confluency. Passage 2 to 6 cells were used for this study, and more than 90% of these cells were positive for CD31 markers.

Positive expression of PECAM-1 (CD31) was analyzed with flow cytometer to ensure the purity of MBECs. Briefly, cells at different passages were trypsinized and labeled with antibody against CD31 (Cat. # 102405; BioLegend, San Diego, CA, USA). Labeled cells were then analyzed with BD FACSCelesta™ flow cytometry (Becton, Dickinson and Company, Franklin Lakes, NJ, USA).

### 2.4. Bacterial Culture and Preparation

*Porphyromonas gingivalis* (*P. gingivalis*; ATCC^®^ 33277™; ATCC; Manassas, VA, USA) was cultured under an anaerobic condition in 37 °C incubator using CDC ANA agar 5% sheep blood (Dr.plate, Taipei, Taiwan) and tryptic soy broth (TSB; Cat. # 7164A; Acumedia, Lansing, MI, USA). Heat-killed and live *P. gingivalis* were freshly prepared for each experiment. Briefly, *P. gingivalis* was centrifuged, washed with phosphate buffer saline (PBS) and dispersed in serum- and antibiotic-free cell culture medium. *P. gingivalis* was applied directly to the cells for live condition while it was treated with heat at 80 °C for 10 min [50] for heat-killed condition.

### 2.5. Bacterial Infection and Antioxidant Treatment

For the following experiments, bEnd.3 and MBECs were infected with heat-killed *P. gingivalis* at the multiplicity of infection (MOI) level of 500 or live *P. gingivalis* at the MOI level of 1, 50, 100, 200 or 500 for 90 min or left uninfected. *P. gingivalis* was discarded. Cells were washed 2 times with PBS. Fresh medium was added to cells and incubated for the specified periods as mentioned in each experiment. To elucidate the effect of antioxidant on *P. gingivalis*-induced inflammatory response and cell death, in a separate experiment, bEnd.3 cells were treated with 5, 10 or 20 mM *N*-Acetyl-L-cysteine (NAC; Cat. # A9165; Sigma-Aldrich, St. Louis, MO, USA) for 2 h before infection with *P. gingivalis*.

### 2.6. Adherence of P. gingivalis to Host Cells

The bEnd.3 cells at 5 × 10^5^ cells/mL were plated onto a 6-well plate and cultivated overnight. Cells were treated with *P. gingivalis* for 90 min. Unbound *P. gingivalis* was washed out from cells. Cells were detached enzymatically from plate and lysed with sterilized deionized water for 30 min. Cell lysates were collected by the centrifugation for 10 min at 5000× *g*. DNA was extracted with a Genomic DNA mini kit (Cat. # GB100/300; Geneaid, New Taipei City, Taiwan) following a manufacturer’s protocol. Relative number of adhered *P. gingivalis* on host cells was measured by quantitative polymerase chain reaction (qPCR). Each well of MicroAmp^®^ optical 96-well reaction plate (Applied Biosystems, Foster City, CA, USA) contained 12.5 μL of Power SYBR^®^ green master mix (Cat. # 4367659; Applied Biosystems, Foster City, CA, USA), 1 μL of forward and reverse primer, 9.5 μL of DI water, and 2 μL of DNA template. Forward and reverse primer of bacterial 16S rDNA for detecting *P. gingivalis* was synthesized by MDBio (Taipei, Taiwan) with the sequences:

5′-AGAGTTTGATCCTGGCTCAG-′3 (forward)

5′-GGCTACCTTGTTACGACTT-′3 (reverse)

Mouse β-actin were used as an endogenous control for bEnd.3 cell model as they uniformly express. Forward and reverse primers of mouse β-actin were synthesized by Genomics BioSci & Tech (New Taipei City, Taiwan). The sequences were as follows:

Mouse β-actin: 5′-CATCCGTAAAGACCTCTATGCCAAC-′3 (forward)

5′-ATGGAGCCACCGATCCACA-’3 (reverse)

The amplification was detected with ABI 7300 Real-Time PCR system (Applied Biosystems, Foster City, CA, USA) with the default thermal cycling conditions. Relative number of adhered *P. gingivalis* to cells was calculated based on the relative quantification method described in Applied Biosystems User Bulletin No. 2 (P/N 4303859B) and [51]. Cycle threshold (C_T_) value of each sample was obtained and normalized with endogenous housekeeping gene (β-actin) to get ∆C_T_. ΔΔC_T_ was obtained after the normalization of ΔC_T _value with an assay calibrator (untreated group). The relative number of adhered *P. gingivalis* to cells was then calculated by $2^{-\Delta \Delta C_{T}}$.

### 2.7. Adherence of FITC-Labeled P. gingivalis to Host Cells

To confirm the adhesion of *P. gingivalis* to host cells, *P. gingivalis* was pre-labeled with FITC before the infection so it is visible under fluorescence microscope once it adheres. The bEnd.3 cells were plated at a density of 5 × 10^4^ cells/mL in a 6-well plate. *P. gingivalis* was pre-labeled with Fluorescein-5-Isothiocyanate (FITC ‘Isomer I’; Cat. # F1906; Life Technologies, Carlsbad, CA, USA) following the methods adapted from [52] and a manufacturer’s protocol. Heat-killed or live *P. gingivalis* was resuspended in 0.5 M sodium bicarbonate (NaHCO_3_; Cat. # S5761; Sigma-Aldrich, St. Louis, MO, USA), pH 8.0 containing FITC at a concentration of 10 mg/mL and incubated under an anaerobic condition in 37 °C incubator with shaking for 60 min. *P. gingivalis* was harvested by centrifugation. Unbounded FITC was washed out from *P. gingivalis*. *P. gingivalis* pre-labeled with FITC was resuspended in serum- and antibiotic- free cell culture medium. Live or heat-killed FITC-labeled *P. gingivalis* was incubated with cells for 90 min. Non-adhered FITC-labeled *P. gingivalis* was removed before the observation with IX71 inverted system microscope (Olympus, Shinjuku, Tokyo, Japan). Photos were taken at the magnification of 20X using Spot software version 4.6 (SPOT Imaging, Sterling Heights, MI, USA).

### 2.8. MTT Assay

Cell viability after *P. gingivalis* infection was determined by a colorimetric method based on a conversion of a yellowish solution, Thiazolyl Blue Tetrazolium Bromide (MTT), by dehydrogenase and reducing agents found in the mitochondria and endoplasmic reticulum to water-insoluble formazan product [53]. The protocol used in this study was adapted from Ko et al. [35]. Briefly, 10,000 cells/mL bEnd.3 and MBECs were cultured on a 24-well plate overnight. After 90 min of *P. gingivalis* infection with or without NAC pretreatment, *P. gingivalis* was discarded from a monolayer of cells. Cells were incubated for an additional 24 h. Then, the supernatant was removed. MTT working solution (30 μL) containing 5 mg/mL of Thiazolyl Blue Tetrazolium Bromide (MTT; Cat. # M5655; Sigma-Aldrich, St. Louis, MO, USA) in PBS and 270 μL of cell culture medium were added to each well. After 1 h of incubation at 37 °C, formazan product was dissolved with dimethyl sulfoxide (DMSO; Cat. # 802912; Merck Millipore, Billerica, MA, USA) and transferred to a 96-well plate. The absorbance was measured by Epoch Microplate Spectrophotometer (BioTek, Winooski, VT, USA) at a wavelength of 540 nm. Number of viable cells after the infection was compared with untreated (control) group. Survival rate is presented as a percentage of control.

### 2.9. Trypan Blue Exclusion Assay

To confirm the results of cell viability after *P. gingivalis* infection, Trypan blue exclusion assay was also done in addition to MTT assay. The bEnd.3 cells at a density of 5 × 10^4^ cells/mL were cultured in a 6-well plate overnight. Cells were infected with *P. gingivalis* with or without NAC pretreatment before replaced with fresh medium and cultured for an additional 24 h. Treated cells were enzymatically detached from well plate and stained with 0.5% Trypan Blue dye solution (Cat. # 03-102-1B; Biological Industries, Cromwell, CT, USA). Cell number was calculated and compared with control group to determine survival rate as a percentage of control.

### 2.10. Nuclear Staining

Nuclear condensation, one hallmark of apoptosis, was observed after *P. gingivalis* infection. The bEnd.3 or MBECs were plated onto a 6-well plate at a concentration of 5 × 10^4^ cells/mL and incubated with *P. gingivalis* with or without pretreatment with NAC. Six, 12, or 24 h after treatment, cells were fixed with 4% paraformaldehyde (Cat. # PB0684; Bio Basic, Toronto, ON, Canada) for 10 min then permeabilized with 0.1% Triton X-100 (Cat. # AT1050-0500; Bionovas Biotechnology, Toronto, ON, Canada) for 10 min at room temperature. Nucleus of fixed cells were stained with 1 μg/mL of 4′,6-diamidino-2-phenylindole, dihydrochloride (DAPI; Cat. # 62247; Thermo Fisher Scientific, Waltham, MA, USA) in PBS for 10 min at room temperature. Nuclear condensation was observed with IX71 inverted system microscope (Olympus, Shinjuku, Tokyo, Japan). Photos were taken at the magnification of 20× using Spot software version 4.6 (SPOT Imaging, Sterling Heights, MI, USA).

### 2.11. FITC Annexin V and PI Staining

Cells at a density of 1 × 10^6^ cells/mL were seeded in a 10-cm (bEnd.3) or 6-cm (MBECs) petri dish and cultured overnight. Various conditions of NAC were used to treat cells as mentioned in “Bacterial infection and antioxidant treatment” section. *P. gingivalis* was allowed to infect cells for 90 min before replaced with fresh medium and cultured for a further 24 h. Cells were enzymatically collected and proceeded with FITC Annexin V apoptosis detection kit (Cat. # 556547; Becton, Dickinson and Company, Franklin Lakes, NJ, USA). Briefly, cells were resuspended with 1XAnnexin V binding buffer. Then, 100 μL of the solution was transferred to 5 mL tube. Five microliters of FITC Annexin V and/or PI were added to the solution and incubated for 15 min in the dark. After the incubation period, 400 μL of 1XAnnexin V binding buffer was added to the solution and analyzed with BD FACSCanto™ II flow cytometry or BD FACSCelesta™ flow cytometry system (Becton, Dickinson and Company, Franklin Lakes, NJ, USA). Unstained cells, cells stained with FITC Annexin V (no PI), and cells stained with PI (no FITC Annexin V) were used to set up compensation and quadrant before acquiring results in each experiment. Cell population of quadrant 2 (FITC Annexin V +/PI −) and quadrant 4 (FITC Annexin V +/PI +) were quantitated and calculated as a percentage of apoptotic cell.

### 2.12. Western Blot Analysis

Expression of NF-κB and inflammatory cytokines protein induced by *P. gingivalis* was identified by Western blot technique following the protocol described previously [54]. To collect total cellular extracts for the detection of inflammatory cytokines, cells were cultured in a 10-cm (bEnd.3) or a 6-cm (MBEC) petri dish at a concentration of 1 × 10^6^ cells/mL and treated with antioxidant and/or *P. gingivalis*. After washed with PBS, fresh medium was added to the cells. After 24 h of incubation, cells were lysed in radioimmunoprecipitation assay (RIPA) lysis buffer (Cat. # 20-188; Merck Millipore, Billerica, MA, USA) with protease inhibitor cocktail (Cat. # P8340; Sigma-Aldrich, St. Louis, MO, USA), and sonicated (10 s/pulse for three pulses) on ice with Misonix Sonicator 3000 Ultrasonic Cell Disruptor (Misonix, Farmingdale, NY, USA). Cell lysates were centrifuged at 13,000× *g* for 5 min at 4 °C. The supernatants were stored for the analysis. Nuclear protein extracts were prepared using nuclear extraction kit (Cat. # ab113474; Abcam, Cambridge, UK). Subsequently, proteins were separated on 8%, 10% or 12% sodium dodecyl sulfate (SDS) polyacrylamide gel and transferred onto Immobilon polyvinylidene fluoride (PVDF) membrane (Cat. # ISEQ00010; Merck Millipore, Billerica, MA, USA). The membranes were blocked with 3% skim milk powder or BSA in Tris-buffered saline and Tween 20 (TBST) for 1 h with agitation. The membranes were then rocked with specific antibodies against IL-1β (Cat. # sc-7884) and β-actin (Cat. # sc-47778) from Santa Cruz Biotechnology (Dallas, TX, USA); TNF-α (Cat. # ab66579) from Abcam (Cambridge, UK); NF-κB p65 (Cat. # GTX102090) and PARP (Cat. # GTX100573) from GeneTex (Irvine, CA, USA) and incubated overnight at 4 °C. Proteins were visualized with Pierce™ ECL Western Blotting Substrate (Cat. # 32106; Thermo Fisher Scientific, Waltham, MA, USA) and observed under BioSpectrum^®^ Imaging System™ (UVP, Upland, CA, USA) or ChemiDoc™ Imaging System (Bio-Rad Laboratories, Hercules, CA, USA). Density of the protein bands was quantified using Image-Pro^®^ Plus software (Media Cybernetics, Rockville, MD, USA).

### 2.13. NF-κB p65 Transcription Factor DNA Binding Activity Assay

The translocation of NF-κB p65 into nucleus after *P. gingivalis* infection was also detected using specific transcription factor DNA binding activity technique in nuclear extracts (NF-κB p65 transcription factor assay kit; Cat. # ab133112; Abcam, Cambridge, UK). The assay was done as outlined in manufacturer’s protocol. Transcription factor binding assay buffer was prepared and added to each well followed by the addition of nuclear extracts that were prepared earlier for Western blot analysis. Plate was sealed and incubated overnight at 4 °C. The buffer was discarded. Then, plate was washed five times with wash buffer before the addition of transcription factor NF-κB p65 primary antibody. Incubated plate for 1 h at room temperature without agitation. At the end of incubation period, plate was washed 5 times with wash buffer and replaced with transcription factor goat anti-rabbit HRP conjugate. After 1 h of incubation at room temperature, plate was washed 5 times and developed with transcription factor developing solution. The plate was kept under agitation at room temperature with the protection from light for 15 to 45 min until the color turned medium or dark blue. Stop solution was added and the absorbance was read at 450 nm. NF-κB p65 DNA-binding activity in a fold of control was calculated.

### 2.14. Measurement of Reactive Oxygen Species Production

The production of intracellular reactive oxygen species (ROS) by bEnd.3 and MBECs after *P. gingivalis* infection was measured by the conversion of DCFH-DA, a cell-permeable non-fluorescent probe, into highly fluorescent DCF upon oxidation as described previously [35,55]. The bEnd.3 or MBECs at a density of 5 × 10^5^ cells/mL were cultured overnight in a T25 flask. Different concentrations of NAC were incubated with cells. After incubation period, NAC was removed. Cells were incubated with 50 μM of DCFH-DA (Cat. # D6883; Sigma-Aldrich, St. Louis, MO, USA) for 30 min followed by incubation with 500 μM of hydrogen peroxide solution (H_2_O_2_; Cat. # 18304; Sigma-Aldrich, St. Louis, MO, USA) or *P. gingivalis* for 90 min. Cells were collected enzymatically and analyzed with BD FACSCanto™ II flow cytometry or BD FACSCelesta™ flow cytometry system (Becton, Dickinson and Company, Franklin Lakes, NJ, USA).

### 2.15. Statistical Analysis

All of the experiments were performed for 3 to 4 replications. The data are shown as the mean ± standard error of the mean (SEM). Statistically significant differences in each study group were analyzed with one-way analysis of variance (ANOVA) and Tukey’s multiple comparison test using Graphpad Prism version 5.03 and 7.0a (Graphpad Software, San Diego, CA, USA). A *p* value less than 0.05 was defined as statistically significant.

## 3. Results

### 3.1. Adherence of P. gingivalis to bEnd.3 Cells Initiated the Infection Process

**Figure 1:**
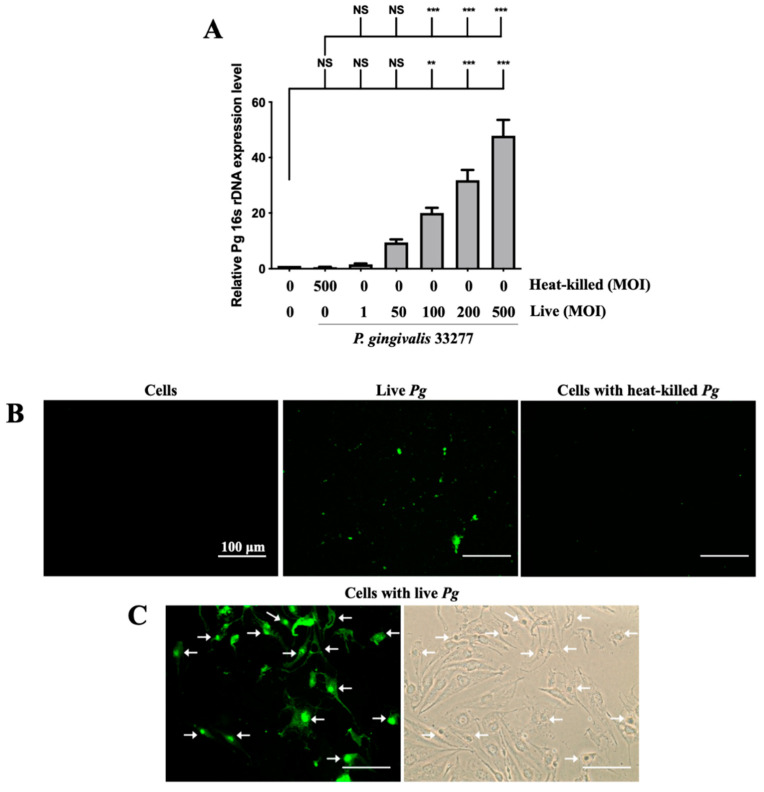
Expression of *P. gingivalis* in brain endothelial cells after bacteria infection. *P. gingivalis* and heat-killed bacteria are pre-labeled with FITC before cell infection. bEnd.3 cells are infected with various MOIs (1, 50, 100, 200 or 500) of *P. gingivalis* or *P. gingivalis* for 90 min, and the expression of *P. gingivalis* is quantitated by qPCR assay with bacterial 16S rDNA (**A**). bEnd.3 cells alone, FITC-labeled live *P. gingivalis* alone, and FITC-labeled heat-killed *P. gingivalis* with cells are observed using a fluorescence microscope (**B**). FITC-labeled *P. gingivalis* cells after bacterial infection are shown in (**C**). Arrows show invasion and attachment of FITC-labeled *P. gingivalis* to infected cells. Data are presented as the mean ± SEM (*n* = 4). **, *p* < 0.01; ***, *p* < 0.001; NS, *p* > 0.05 indicate a significant difference compared to the heat-killed *P. gingivalis* and control groups. The scale bar represents 100 μm (**B**).

Adherence of pathogenic bacteria to host cells is a crucial step for initializing and progressing the infection [56]. Infection begins with the attachment of *P. gingivalis* to host cells via the fimbriae; when bacterial proteases and lipopolysaccharide (LPS) come into contact, they damage supportive periodontal tissues, resulting in a severe inflammatory response [57]. The expression of *P. gingivalis* in brain endothelial cells was determined by the levels of 16s rDNA through qPCR. As shown in Figure 1A, the expression of 16s rDNA increased in a dose-dependent manner from 1-fold to 50-fold after *P. gingivalis* infection. Moreover, the increase in *P. gingivalis* expression in infected cells was evident at a higher multiplicity of infection (MOI) level (100 to 500) (Figure 1A). However, heat-killed *P. gingivalis* did not show any bacterial DNA expression. Furthermore, we used FITC-labeled *P. gingivalis* to view the infected bacteria in the brain endothelial cells. As shown in Figure 1B, live *P. gingivalis* pre-labeled with FITC was observed under a fluorescence microscope, but not cells alone or heat-killed *P. gingivalis.* Moreover, the *P. gingivalis* pre-labeled with FITC was observed to invade host cells (Figure 1C). The strong intensity of green fluorescence also indicated that the *P. gingivalis* pre-labeled with FITC were aggregated in nuclei (indicated by arrows; Figure 1C). These findings suggest that only live *P. gingivalis* has the ability to infect the brain endothelial cells, and the infected bacteria adhere and invade the host cells.

### 3.2. P. gingivalis Caused the Alteration of Cell Morphology and Cell Viability in Brain Endothelial Cells

**Figure 2:**
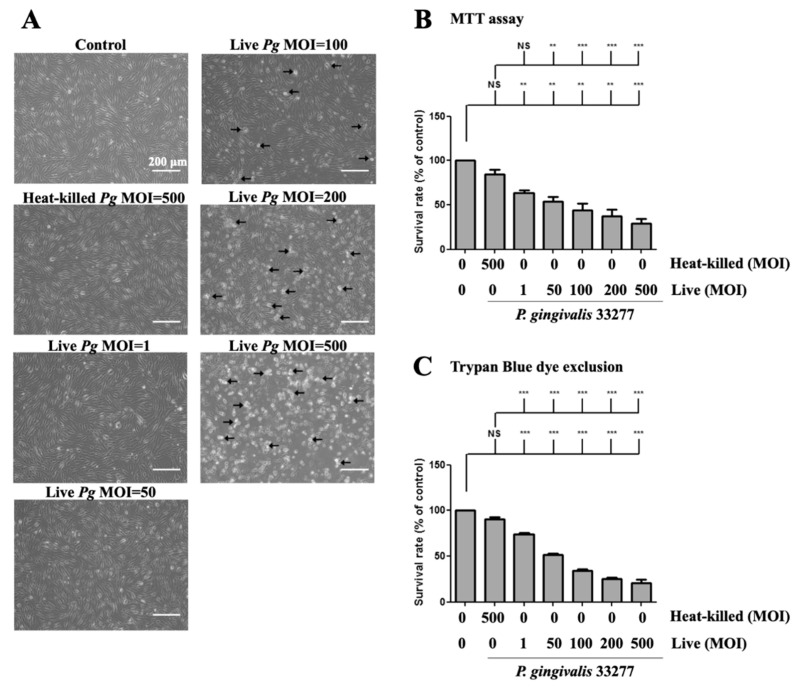
*P. gingivalis* caused cell morphological changes and cell death in brain endothelial cells. bEnd.3 cells are infected with live (1, 50, 100, 200 or 500 MOI levels) or heat-killed (500 MOI level) *P. gingivalis* for 90 min and further incubated with fresh medium for another 24 h. (**A**) Cell morphology is observed with a light microscope. The arrows indicate the death cells. The scale bar represents 200 μm. The cell survival rate is determined by the MTT assay (**B**) and Trypan Blue exclusion test (**C**) and calculated as the percentage of control. Data are presented as mean values ± SEM (*n* = 4). **, *p* < 0.01; ***, *p* < 0.001; NS, *p* > 0.05 indicate significant differences compared to the control or heat-killed *P. gingivalis* group.

Next, we determined whether *P. gingivalis* infection affects cell morphology and cell viability. The cell survival rate after *P. gingivalis* infection was determined using a microscope and MTT assay, respectively. Increased *P. gingivalis* levels from 100 to 500 MOI showed increased detachment of brain endothelial cells, when observed under the microscope. (Figure 2A). Remarkably, a notable change in morphology, such as shrinkage and increased detachment from the culture plate, was observed at MOI levels of 200 and 500 (Figure 2A). In addition, either low doses (MOI 1 and 50) of live bacteria or high dose of heat-killed (MOI 500) bacteria only observed a few detached cells and did not show morphological changes (Figure 2A). Moreover, MTT assay showed that cell viability was significantly decreased by *P. gingivalis* infection in a dose-dependent manner (Figure 2B). Heat-killed *P. gingivalis* did not affect cell viability (Figure 2B). We further used the trypan blue exclusion assay to confirm the results of the MTT assay. As shown in Figure 2C, the cell survival rate was significantly decreased by *P. gingivalis* infection by 30% to 70%. There was no significant difference in cell viability following heat-killed *P. gingivalis* treatment (Figure 2C). In addition, *P. gingivalis* infection did not affect cell viability in the astrocyte cells (Figure S1A,B). These results suggest that infection with live *P. gingivalis*, but not with heat-killed bacteria, specifically induces cell death in brain endothelial cells.

### 3.3. Infection of P. gingivalis Induces Cell Apoptosis in Brain Endothelial Cells

**Figure 3:**
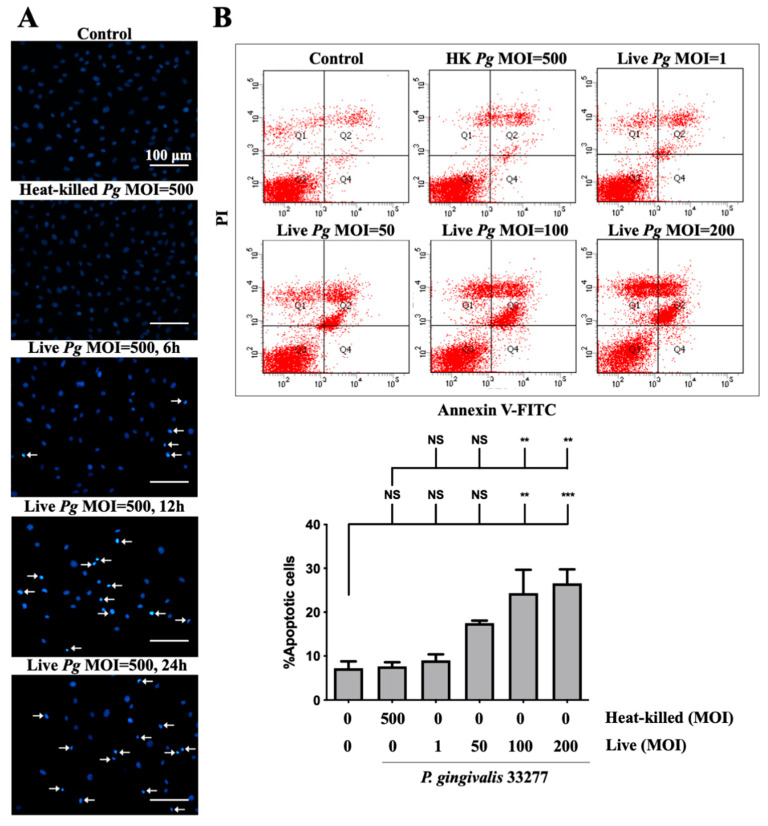
*P. gingivalis* infection induces cell apoptosis in bEnd.3 brain endothelial cells. (**A**) Cells are infected with either heat-killed or live *P. gingivalis* at an MOI of 500 for 90 min. After being replaced with fresh medium for 6, 12, or 24 h, nuclear condensation is determined by DAPI staining. Arrows indicate cells with a condensed nucleus. The scale bar represents 100 μm. (**B**) Cells are treated with various live bacteria (1, 50, 100 and 200 MOI) or heat-killed bacteria (500 MOI) for 90 min. Cell survival is stained with Annexin V FITC/PI after 24 h and analyzed with a flow cytometer (upper panel). The quantitative percentage of apoptotic cells is shown in the lower panel. Data are presented as mean values ± SEM (*n* = 4). Significant differences of the control or heat-killed *P. gingivalis* group are expressed as **, *p* < 0.01; ***, *p* < 0.001; NS, *p* > 0.05.

Previous studies reported on the induction of cell apoptosis by infection with *P. gingivalis* in human aortic endothelial cells [13] and gingival epithelial cells [58]. Thus, we determined whether *P. gingivalis* infection caused apoptosis in brain endothelial cells. As shown in Figure 3A, a significant number of condensed nuclei were observed after *P. gingivalis* infection, which was initiated at 90 min and reached a peak at 12 h and was sustained for 24 h. No condensed nucleus was observed in cells infected by heat-killed bacteria (Figure 3A). We further performed the staining of annexin V and propidium iodide (PI) to determine apoptotic cells following treatment with *P. gingivalis* in endothelial cells. Cells infected with low MOI *P. gingivalis* (50 MOI) slightly induced apoptosis (Figure 3B). Importantly, the number of apoptotic cells was significantly elevated after infection with MOI (100 and 200 MOI). However, the number of apoptotic cells was not observed in either heat-killed or live *P. gingivalis* at an MOI of 1 (Figure 3B). These results indicate that infection with *P. gingivalis* causes cell death by inducing apoptosis in brain endothelial cells.

### 3.4. Increased Proinflammatory Cytokines TNF-α and IL-1β Expression in P. gingivalis Activated Brain Endothelial Cells

**Figure 4:**
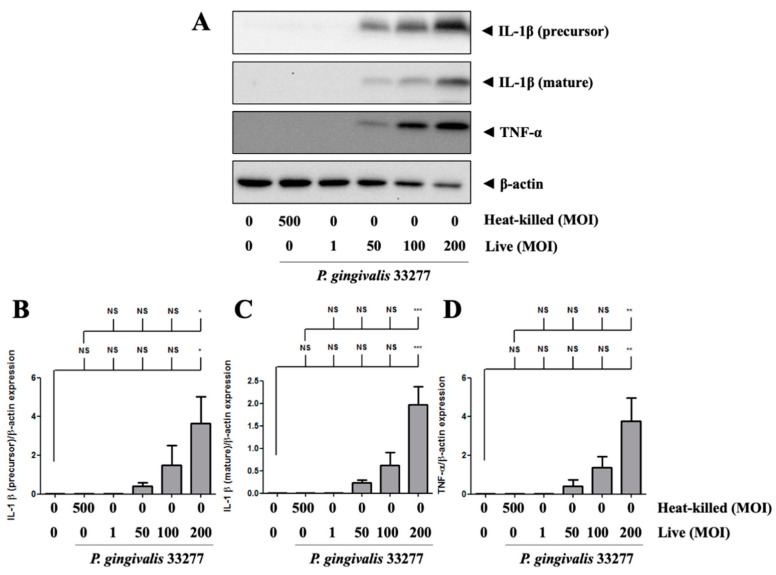
*P. gingivalis* up-regulated IL-1β and TNF-α protein levels in bEnd.3 cells. (**A**) Cells are infected with live (MOI 1, 50, 100 and 200) or heat-killed (MOI 500) *P. gingivalis* for 90-min followed by 24-h fresh medium incubation. Protein expressions of IL-1β (precursor), IL-1β (mature), TNF-α, and β-actin are determined using Western blot analysis. The quantitative results were shown in bar graph (**B**–**D**). Data are presented as mean values ± SEM (*n* = 3). Significance differences of the control or heat-killed *P. gingivalis* group are expressed as *, *p* < 0.05; **, *p* < 0.01; ***, *p* < 0.001 and NS, *p* > 0.05.

*P. gingivalis* increased the precursor form of IL-1β protein expression in brain endothelial cells in a dose-dependent manner (Figure 4A). In particular, the mature form of IL-1β expression was also observed in *P. gingivalis*-infected brain endothelial cells in a dose-dependent manner (Figure 4A). *P. gingivalis* also activated TNF-α protein expression in brain endothelial cells in a dose-dependent manner (Figure 4A,D). Moreover, heat-killed *P. gingivalis* did not affect IL-1β and TNF-α protein expression (Figure 4A–D). These results suggest that only live *P. gingivalis* can infect brain endothelial cells and induce proinflammatory cytokine expression.

### 3.5. P. gingivalis Elevated ROS Production and Up-Regulated NF-κB p65 Activation

**Figure 5:**
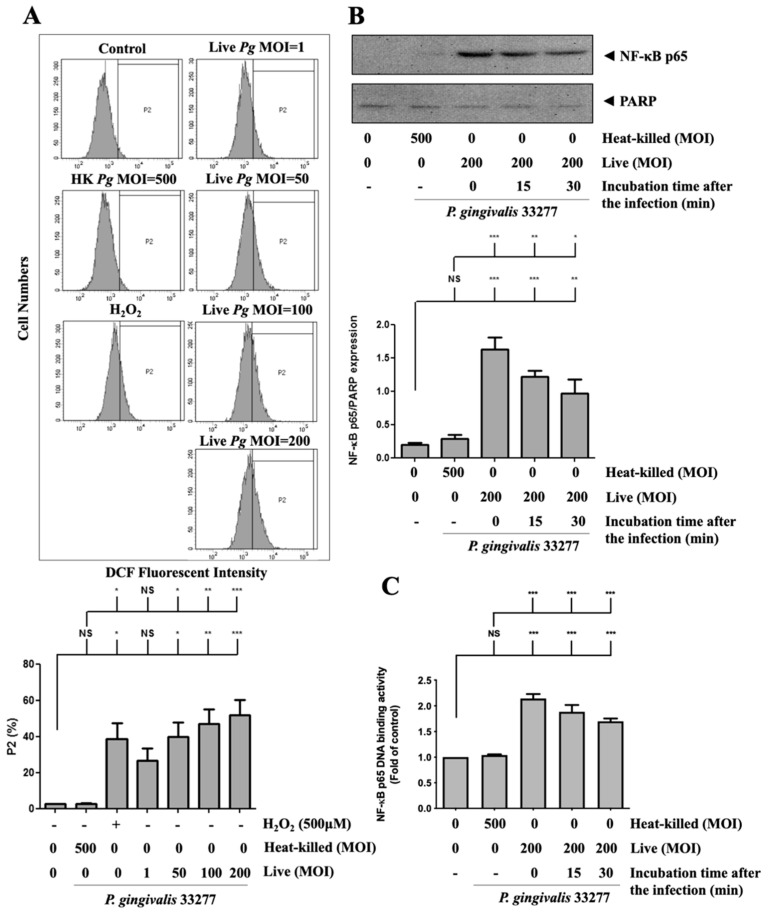
*P. gingivalis* stimulated the production of reactive oxygen species (ROS) and increased NF-κB activation in brain endothelial cells. (**A**) bEnd.3 cells are pre-incubated with DCFH-DA (50 μM) for 30-min. After several washes, cells are treated with various MOI (1, 50, 100, or 200) of *P. gingivalis* or heat-killed bacteria (MOI of 500) for 90-min. ROS production is determined by the DCF fluorescence intensity using a flow cytometer. Treatment with hydrogen peroxide (H_2_O_2_; 500 μM) is a positive control. The quantitative percentage of the P2 area is shown in a low panel. bEnd.3 cells are infected with *P. gingivalis* (200 MOI) for 90 min followed by further incubation with fresh medium for the indicated time periods (15 or 30 min). The nuclear fraction of NF-κB p65 protein is determined by Western blotting analysis (**B**). The NF-κB p65 DNA-binding activity was determined by the transcription factor binding assay (**C**). Data in the bar graph are presented as mean values ± SEM (*n* = 4). Significant differences of the control and heat-killed *P. gingivalis* groups are expressed as *, *p* < 0.05; **, *p* < 0.01; ***, *p* < 0.001; NS, *p* > 0.05.

Next, to investigate whether ROS production is involved in *P. gingivalis*-induced endothelial cell death, the intracellular ROS level was determined after *P. gingivalis* infection. As shown in Figure 5A, *P. gingivalis* enhanced ROS production by approximately 15-to 25-fold in infected brain endothelial cells. We have previously reported that activation of NF-κB is a critical modulator of the up-regulation of inflammatory cytokines [59,60,61]. As shown in Figure 5B, *P. gingivalis* induced a significant translocation of NF-κB p65 protein to the nucleus. After 90 min of *P. gingivalis* infection, the nuclear NF-κB p65 protein increased immediately and was sustained for 30 min. In addition, the NF-κB p65 DNA-binding activity increased approximately 2.1-fold immediately and was sustained for 30 min after *P. gingivalis* exposure (Figure 5C). In addition, heat-killed bacteria did not affect ROS production or activation of NF-κB p65. Our results indicate that *P. gingivalis* infection increases ROS production and NF-κB activation in brain endothelial cells.

### 3.6. P. gingivalis-Induced ROS Production Regulates Proinflammatory Cytokine Expression and Cell Death

**Figure 6:**
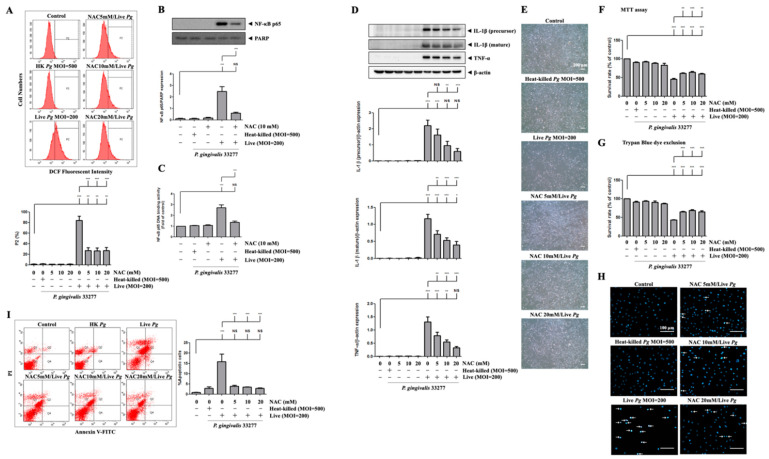
Inhibition of *P. gingivalis-medicated ROS production* inhibits NF-κB activation, proinflammatory cytokine expression, cell apoptosis, and cell death in bEnd.3 cells. Cells are treated with various concentrations (0, 5, 10 or 20 mM) of NAC for 2 h and then infected with *P. gingivalis* (200 MOI) for 90 min; the ROS production is determined by DCF fluorescence using a flow cytometer (**A**). Total protein is collected, and the expression of IL-1β (precursor), IL-1β (mature), and TNF-α are determined by Western blotting (**D**). Morphological alterations are observed under a light microscope (**E**). The cell survival rate is evaluated by MTT assay (**F**) and Trypan Blue exclusion test (**G**). The nuclear condensation is performed by DAPI staining and viewed with a fluorescence microscope (**H**). Apoptotic cells are determined by Annexin V/PI staining and quantitated by a flow cytometer (**I**). Cells were treated with NAC (10 mM) and infected with *P. gingivalis*, the nuclear NF-κB p65 and PARP protein expression are determined by Western blot analysis (**B**), and transcriptional factor binding activity is determined by NF-κB p65 DNA-binding activity (**C**). Data are presented as mean values ± SEM (*n* = 4). Significant differences of the control group and groups with or without NAC treatment are expressed as *, *p* < 0.05; **, *p* < 0.01; ***, *p* < 0.001; NS, *p* > 0.05. Scale bars are 200 μm in (**E**) and 100 μm in (**H**).

We then examined whether *P. gingivalis*-induced ROS production modulates NF-κB activation, proinflammatory cytokine expression, cell apoptosis, and endothelial cell death. Previously, we reported that NAC neutralized ROS production and protected against cell death [62]. Figure 6A shows that treatment with NAC significantly reduced ROS production in *P. gingivalis*-infected cells by almost 70%. Moreover, treatment with NAC decreased the nuclear translocation of NF-κB p65 by approximately 70% after *P. gingivalis* infection (Figure 6B). Similarly, NAC treatment also drastically decreased NF-κB p65 DNA-binding activity by 70% compared to infected cells alone (Figure 6C). Accordingly, NAC treatment noticeably decreased the protein levels of IL-1β (precursor), IL-1β (mature), and TNF-α in *P. gingivalis-infected* cells in a dose-dependent manner (Figure 6D). We further evaluated whether the bacterial infection-altered endothelial cell morphology was reversed by NAC treatment. As shown in Figure 6E, NAC treatment clearly showed a greater number of intact cells following *P. gingivalis* inoculation. Remarkably, *P. gingivalis*-induced cell death was also rescued by NAC treatment (Figure 6F,G). In addition, NAC treatment alone at concentrations of up to 20 mM had no toxicity on bEnd.3 cells (Figure 6F,G). Treatment with NAC improved the cell survival rate from 30% to 50% following bacterial infection. Furthermore, treatment with NAC also dramatically reduced *P. gingivalis*-induced condensed nuclei (Figure 6H) and apoptotic death of brain endothelial cells (Figure 6I). Importantly, NAC significantly reduced the percentage of apoptotic cells by approximately 80% (Figure 6I). These results suggest that *P. gingivalis* infection induces brain endothelial cell apoptosis and cell death through the expression of ROS/NF-κB/proinflammatory cytokines.

### 3.7. Infection with P. gingivalis in Primary Mouse Brain Endothelial Cells (MBECs) Elevates ROS/NF-κB Activation and Results in Proinflammatory Cytokine Expression and Cell Death

**Figure 7:**
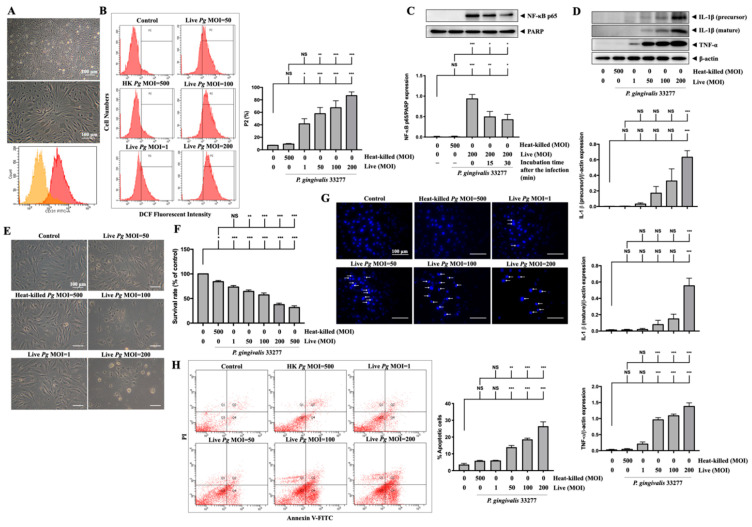
*P. gingivalis* up-regulates ROS and NF-κB activation and increases the expression of inflammatory cytokines and cell death in primary mouse brain endothelial cells (MBECs). Cells are obtained from 6–7 weeks of adult C57BL/6 mice. The morphology of MBECs at passage 1 is observed with a light microscope ((**A**), upper panel). The expression of platelet endothelial cell adhesion molecule 1 (PECAM-1/CD31) is examined by flow cytometry and presented as histograms ((**A**), lower panel). (**B**) MBECs are infected with various MOI (1, 50, 100 and 200) of *P. gingivalis* for 90 min. The intracellular ROS is determined by DCF fluorescence intensity using flow cytometry shown as gated histogram (**B**). Followed by 24 h of incubation, the expression of IL-1β (precursor), IL-1β (mature), TNF-α, and β-actin proteins are determined by Western blot analysis. Quantitative results are presented as bar graphs in low panels (**D**). Cell morphology is observed under a light microscope (**E**). Condensed nuclei are determined by DAPI staining and viewed with a fluorescence microscope. Arrows indicated condensed nuclei (**G**). Annexin V/PI staining is performed to determine apoptotic cells and analyzed by a flow cytometer (**H**). (**C**) Cells are infected with *P. gingivalis* for 90-min and incubated with fresh medium for the indicated time periods (15 and 30 min), nuclear NF-κB p65 translocation is determined by Western blot analysis. (**F**) MBECs are infected with various MOI (1, 50, 100, 200 and 500) of *P. gingivalis* for 90 min. The cell viability is determined by MTT assay (as a percentage of control) after 24 h of incubation. Data are presented as mean values ± SEM (*n* = 4). Significant differences of the control group and heat-killed *P. gingivalis* groups are expressed as *, *p* < 0.05; **, *p* < 0.01; ***, *p* < 0.001; NS, *p* > 0.05. Scale bars are 200 and 100 μm in (**A**) and 100 μm in (**E**,**G**).

Next, we used primary MBECs isolated from adult C57BL/6 mice to further confirm the effects of *P. gingivalis-*induced oxidative stress and cell death. As shown in Figure 7A, the morphology of the untreated isolated cells is exhibited. At passage 1, the majority of cells (over 90%) positively expressed platelet endothelial cell adhesion molecule 1 (PECAM-1/CD31) using flow cytometry analysis (Figure 7A). MBECs were stained with DCFH-DA for both untreated and treated with live or heat-killed bacteria at various MOI levels for 90 min, and the fluorescent product DCF was measured by flow cytometry (Figure 7B). Oxidative stress due to elevated ROS, caused by *P. gingivalis* infection, increased the fluorescent product DCF. As a result, oxidized cells populated P2 (Figure 7B). Dose-dependent (1 to 200 MOIs) infection of live *P. gingivalis* in MBECs increased the percentage of the P2 cell population from 7% in the control group to 90% in the live *P. gingivalis* MOI 200 group (Figure 7B). NF-κB p65 protein expression was also identified in nuclear extracts of MBECs treated with live bacteria (MOI 200) for 90 min and then incubated for another 0, 15, or 30 min. The levels of the NF-κB p65 protein peaked at 90 min of infection with *P. gingivalis*, as shown in Figure 7C, and then began to decrease at 15 and 30 min of continued incubation. Moreover, 24 h after infection, we detected the overexpression of IL-1β (precursor) and IL-1β (mature) proteins in cells treated with live bacteria at MOI 50 upwards and TNF-α protein overexpression exhibited at MOI 1 and above (Figure 7D). Live *P. gingivalis* at an MOI of 50 upwards changed in morphology as the cells shrank and detached from the plate (Figure 7E) and decreased the survival rate of MBECs from 100% to 64% at MOI 50, 57% at MOI 100, and 37% at MOI 200, as evaluated by the MTT assay (Figure 7F). Twenty-four hours after *P. gingivalis* infection, we stained the cell nucleus with DAPI dye and detected numerous condensed nuclei, representing apoptosis in infected cells at over MOI 50, as indicated by the white arrow in Figure 7G. In line with DAPI staining, the results of Annexin V and PI staining indicated significant apoptotic cells inoculated with live *P. gingivalis* at MOI levels of 50 upwards (Figure 7H).

## 4. Discussion

Although some case-control studies and epidemiologic studies reported an increased risk of stroke due to periodontal disease [63], little is known about the underlying mechanisms. A recent study reported that oral *P. gingivalis* infection in mice resulted in colonization and increased β-amyloid plaque production in the brain [19]. *P. gingivalis* infection causes neuroinflammation and cognitive impairment in middle-aged mice [64]. Furthermore, gingipain inhibition reduced neuroinflammation and rescued neurons in the hippocampus in the bacterial load of a *P. gingivalis* brain infection model [19]. Importantly, periodontitis is associated with an increase in cognitive decline in AD that has been attributed to systemic inflammation [5]. An oral gingipain inhibitor is now being tested in a clinical study of patients with early AD [26]. In addition, the virulence factors of *P. gingivalis* in periodontitis, such as gingipain, cause apoptotic death in gingival epithelial cells [65,66] and fibroblast cells [67]. Previous reports also found that *P. gingivalis* causes apoptotic death in vascular endothelial cells [68,69]. Accumulating studies have discovered that the concept of the oral-brain axis facilitates the development of novel biomarkers of brain diseases by contributing diagnostic and therapeutic strategies [70]. The present study supports previous studies showing that *P. gingivalis* infection causes apoptotic cell death in bEnd.3 brain endothelial cells and primary MBECs, suggesting a link between periodontal infection and brain vascular damage.

*P. gingivalis* adherence to host cells is a crucial step in the initiation and progression of chronic periodontitis [71]. Bacterial surface components such as fimbria and gingipain have been found to mediate adherence, invasion, colonization, and immune defense evasion of bacteria to host cells [72,73,74,75]. The results of FITC-labeled *P. gingivalis* adherence to brain endothelial cells (bEnd.3) together with the relative levels of bacterial expression derived from our qPCR study showed that live *P. gingivalis* initially adhered and started invading host cells, whereas heat-killed *P. gingivalis* did not. Since bacterial components such as fimbriae and gingipain were found to facilitate the adherence and invasion of host cells, it is possible that heat-killed *P. gingivalis* depleted these components during heat treatment, thus losing its ability to adhere and invade host cells. Previous studies have shown that elevated temperature leads to the down-regulation of genes that encode fimbrial proteins [76,77]. Therefore, it could conceivably be hypothesized that subsequent inflammatory responses may not occur in cells infected with heat-killed *P. gingivalis* due to the loss of adherence ability to invade host cells. Our results showed that live but not heat-killed *P. gingivalis* adhered to brain endothelial cells, and only up to 100 MOI levels have a significantly deleterious effect on cell morphology and cell death, which is consistent with previous findings [13,58].

Inflammation has been reported to contribute to the pathogenesis of ischemic brain damage and periodontal inflammation [3,78]. NF-κB belongs to the family of transcription factors that regulate inflammation and immunity in response to pathogens [79]. We have reported that inhibition of NF-κB reduces inflammation mediator expression and prevents myocardial ischemia-reperfusion injury [80]. In addition, our previous findings also reported that NF-κB is a key modulator of IL-1β and TNF-α expression upon stimulation of peptidoglycan, a cell wall component of the Gram-positive bacterium, in the neuroinflammatory response [60]. Proinflammatory cytokines IL-1β and TNF-α are significantly produced during ischemic brain damage by activated cells, such as endothelial cells [81,82], impairing the BBB [83]. IL-1β [84] and TNF-α [85] have been recognized as important vascular and systemic inflammatory mediators that contribute to atherogenesis. Moreover, the administration of IL-1β and TNFα induces cell death in astrocytes [86]. *P. gingivalis* can induce an inflammatory response and up-regulate various cytokines in various cell models [87,88]. Remarkably, *P. gingivalis* infection caused TNF-α and IL-1β expression in the brain tissues, resulting in cognitive impairment in middle-aged mice [60]. Our findings provide valuable information that *P. gingivalis-infected* brain endothelial cells promote NF-κB p65 nuclear translocation, which subsequently overexpress IL-1β and TNF-α, which may progress or aggravate brain vascular diseases.

ROS are required for *P. gingivalis*-induced up-regulation of IL-1β in gingival epithelial cells [89]. Importantly, beta-amyloid induces IL-1β expression through ROS production in microglia [90]. ROS influence NF-κB activation through phosphorylation of IκBα, which in turn affects the DNA-binding activity of NF-κB [91]. The ROS/NF-κB signaling pathway has been found to induce IL-1β [92] and TNF-α [86] induces pathology and cell death. Interestingly, the ROS/NF-κB signaling pathway has been found to induce IL-1β expression and pyroptosis [93]. We previously reported that ROS regulated the activation of caspase-9 and caspase-3 mediated cell apoptosis [94]. Numerous studies have reported that antioxidant therapy with ROS scavenger NAC may be useful in blunting the inflammatory response to various bacteria-infected inflammatory responses. NAC was observed to dramatically inhibit ROS-associated NF-κB activation and cell death [93]. Our previous study also found that ROS production causes cell apoptosis, and treatment with NAC attenuates cell death [62]. Importantly, a previous study has reported that NAC prevents bacterially mediated ROS induction, leading to BBB disruption [95]. Systemic treatment with NAC in infected mice attenuated chemokine secretion in a *Rickettsia conorii* infection mouse model [96]. Importantly, the administration of NAC in patients with sepsis results in decreased NF-κB activation and reduced expression of plasma IL-8 [97]. Our results support these previous findings that treatment with NAC effectively reduced *P. gingivalis-*induced ROS production, cell apoptosis, and brain endothelial cell death.

Although the murine brain endothelial cell line bEnd.3 has been widely used for neurovascular research, cerebral endothelial cell models, and BBB model [98,99,100], the primary culture of adult brain endothelial cells offers better characteristics of brain endothelial cells. Primary adult brain cells are well established to retain most of their biological properties in brain endothelial cells [101]. This study investigated the regulatory mechanism of *P. gingivalis* that infects brain endothelial cells and further determined adult MBECs to better understand the pathophysiological function between periodontal infection and brain vascular damage.

## 5. Conclusions

**Figure 8:**
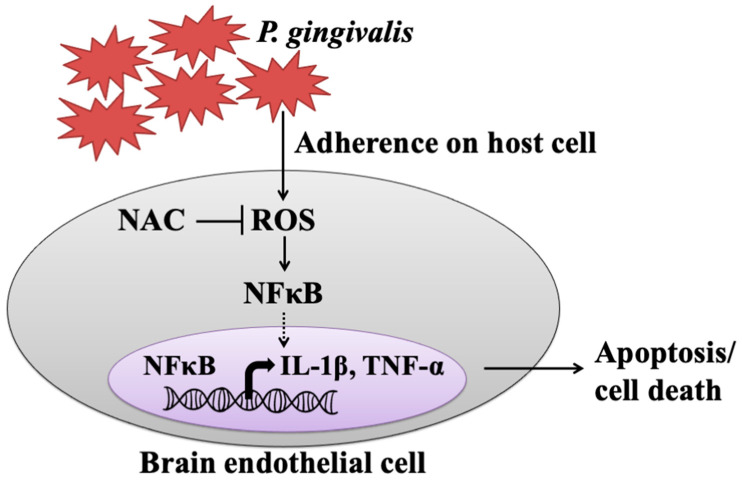
The proposed model shows how *P. gingivalis* induces brain endothelial cell death and that is rescued by NAC. This study provided the possible pathogenesis caused by a periodontal pathogen, *P. gingivalis*, through elevated inflammatory cytokines (IL-1β and TNF-α) and triggered brain endothelial cell death. We found that live *P. gingivalis* greatly adhered to brain endothelial cells caused increased ROS production, which subsequently promoted NF-κB activation, resulting in elevated levels of IL-1β and TNF-α, eventually leading to the death of brain endothelial cells. Additionally, the treatment of the antioxidant NAC attenuates the production of ROS in *P.* gingivalis-infected cells and rescues cell apoptosis and brain endothelial cell death.

The present study showed that live but not heat-killed *P. gingivalis* induces cell apoptosis and progresses to cell death in brain endothelial cells. In addition, infection with *P. gingivalis* also up-regulates the ROS/NF-κB p65 signaling pathway, leading to the expression of IL-1β and TNF-α. Furthermore, neutralization of ROS by the antioxidant NAC effectively attenuates *P. gingivalis*-induced NF-κB activation, expression of proinflammatory cytokines, cell apoptosis, and cell death (Figure 8).

## Data Availability

Data are available from the corresponding author on reasonable request.

## Supplementary Materials

The following are available online at https://www.mdpi.com/article/10.3390/cells10113033/s1. Figure S1: Cell viability of *P. gingivalis*-infected astrocytes.

## References

[1] Petersen P.E., Ogawa H. (2012). The global burden of periodontal disease: Towards integration with chronic disease prevention and control. Periodontology 2000.

[2] Kinane D.F., Stathopoulou P.G., Papapanou P.N. (2017). Periodontal diseases. Nat. Rev. Dis. Primers.

[3] Pihlstrom B.L., Michalowicz B.S., Johnson N.W. (2005). Periodontal diseases. Lancet.

[4] Forner L., Larsen T., Kilian M., Holmstrup P. (2006). Incidence of bacteremia after chewing, tooth brushing and scaling in individuals with periodontal inflammation. J. Clin. Periodontol..

[5] Ide M., Harris M., Stevens A., Sussams R., Hopkins V., Culliford D., Fuller J., Ibbett P., Raybould R., Thomas R. (2016). Periodontitis and cognitive decline in alzheimer’s disease. PLoS ONE.

[6] Sumpio B.E., Riley J.T., Dardik A. (2002). Cells in focus: Endothelial cell. Int. J. Biochem. Cell Biol..

[7] Kozarov E.V., Dorn B.R., Shelburne C.E., Dunn W.A., Progulske-Fox A. (2005). Human atherosclerotic plaque contains viable invasive actinobacillus actinomycetemcomitans and porphyromonas gingivalis. Arterioscler. Thromb. Vasc. Biol..

[8] Aarabi G., Thomalla G., Heydecke G., Seedorf U. (2019). Chronic oral infection: An emerging risk factor of cerebral small vessel disease. Oral Dis..

[9] Hajishengallis G. (2015). Periodontitis: From microbial immune subversion to systemic inflammation. Nat. Rev. Immunol..

[10] Ballini A., Tetè S., Scattarella A., Cantore S., Mastrangelo F., Papa F., Nardi G.M., Perillo L., Crincoli V., Gherlone E. (2010). The role of anti-cyclic citrullinated peptide antibody in periodontal disease. Int. J. Immunopathol. Pharmacol..

[11] Bullon P., Cordero M.D., Quiles J.L., Morillo J.M., Ramirez-Tortosa M.d.C., Battino M. (2011). Mitochondrial dysfunction promoted by porphyromonas gingivalis lipopolysaccharide as a possible link between cardiovascular disease and periodontitis. Free. Radic. Biol. Med..

[12] Miyajima S.-i., Naruse K., Kobayashi Y., Nakamura N., Nishikawa T., Adachi K., Suzuki Y., Kikuchi T., Mitani A., Mizutani M. (2014). Periodontitis-activated monocytes/macrophages cause aortic inflammation. Sci. Rep..

[13] Roth G.A., Ankersmit H.J., Brown V.B., Papapanou P.N., Schmidt A.M., Lalla E. (2007). Porphyromonas gingivalis infection and cell death in human aortic endothelial cells. FEMS Microbiol. Lett..

[14] Yuan H., Zelkha S., Burkatovskaya M., Gupte R., Leeman S.E., Amar S. (2013). Pivotal role of nod2 in inflammatory processes affecting atherosclerosis and periodontal bone loss. Proc. Natl. Acad. Sci. USA.

[15] Hussain M., Stover C.M., Dupont A.P. (2015). Gingivalis in periodontal disease and atherosclerosis–scenes of action for antimicrobial peptides and complement. Front. Immunol..

[16] Kebschull M., Demmer R.T., Papapanou P.N. (2010). “Gum bug, leave my heart alone!”--epidemiologic and mechanistic evidence linking periodontal infections and atherosclerosis. J. Dent. Res..

[17] D’Aiuto F., Nibali L., Parkar M., Suvan J., Tonetti M.S. (2005). Short-term effects of intensive periodontal therapy on serum inflammatory markers and cholesterol. J. Dent. Res..

[18] Tonetti M.S., D’Aiuto F., Nibali L., Donald A., Storry C., Parkar M., Suvan J., Hingorani A.D., Vallance P., Deanfield J. (2007). Treatment of periodontitis and endothelial function. N. Engl. J. Med..

[19] Dominy S.S., Lynch C., Ermini F., Benedyk M., Marczyk A., Konradi A., Nguyen M., Haditsch U., Raha D., Griffin C. (2019). Porphyromonas gingivalis in alzheimer’s disease brains: Evidence for disease causation and treatment with small-molecule inhibitors. Sci. Adv..

[20] Singhrao S.K., Olsen I. (2019). Assessing the role of porphyromonas gingivalis in periodontitis to determine a causative relationship with alzheimer’s disease. J. Oral Microbiol..

[21] Palm F., Lahdentausta L., Sorsa T., Tervahartiala T., Gokel P., Buggle F., Safer A., Becher H., Grau A.J., Pussinen P. (2014). Biomarkers of periodontitis and inflammation in ischemic stroke: A case-control study. Innate. Immun..

[22] Pussinen P.J., Alfthan G., Jousilahti P., Paju S., Tuomilehto J. (2007). Systemic exposure to porphyromonas gingivalis predicts incident stroke. Atherosclerosis.

[23] Pussinen P.J., Alfthan G., Rissanen H., Reunanen A., Asikainen S., Knekt P. (2004). Antibodies to periodontal pathogens and stroke risk. Stroke.

[24] Ghizoni J.S., Taveira L.A., Garlet G.P., Ghizoni M.F., Pereira J.R., Dionísio T.J., Brozoski D.T., Santos C.F., Sant’Ana A.C. (2012). Increased levels of porphyromonas gingivalis are associated with ischemic and hemorrhagic cerebrovascular disease in humans: An in vivo study. J. Appl. Oral Sci..

[25] Hashioka S., Inoue K., Hayashida M., Wake R., Oh-Nishi A., Miyaoka T. (2018). Implications of systemic inflammation and periodontitis for major depression. Front. Neurosci..

[26] Nakanishi H., Nonaka S., Wu Z. (2020). Microglial cathepsin b and porphyromonas gingivalis gingipains as potential therapeutic targets for sporadic alzheimer’s disease. CNS Neurol. Disord.-Drug Targets.

[27] Haditsch U., Roth T., Rodriguez L., Hancock S., Cecere T., Nguyen M., Arastu-Kapur S., Broce S., Raha D., Lynch C.C. (2020). Alzheimer’s disease-like neurodegeneration in porphyromonas gingivalis infected neurons with persistent expression of active gingipains. J. Alzheimer’s Dis. JAD.

[28] Banerjee S., Bhat M.A. (2007). Neuron-glial interactions in blood-brain barrier formation. Annu. Rev. Neurosci..

[29] Lu D.Y., Yu W.H., Yeh W.L., Tang C.H., Leung Y.M., Wong K.L., Chen Y.F., Lai C.H., Fu W.M. (2009). Hypoxia-induced matrix metalloproteinase-13 expression in astrocytes enhances permeability of brain endothelial cells. J. Cell Physiol..

[30] Yeh W.L., Lu D.Y., Lin C.J., Liou H.C., Fu W.M. (2007). Inhibition of hypoxia-induced increase of blood-brain barrier permeability by yc-1 through the antagonism of hif-1alpha accumulation and vegf expression. Mol. Pharmacol..

[31] Naik E., Dixit V.M. (2011). Mitochondrial reactive oxygen species drive proinflammatory cytokine production. J. Exp. Med..

[32] Jayaprakash K., Demirel I., Khalaf H., Bengtsson T. (2018). Porphyromonas gingivalis-induced inflammatory responses in thp1 cells are altered by native and modified low-density lipoproteins in a strain-dependent manner. Apmis.

[33] Lian D., Dai L., Xie Z., Zhou X., Liu X., Zhang Y., Huang Y., Chen Y. (2018). Periodontal ligament fibroblasts migration injury via ros/txnip/nlrp3 inflammasome pathway with porphyromonas gingivalis lipopolysaccharide. Mol. Immunol..

[34] Aguilera G., Colín-González A.L., Rangel-López E., Chavarría A., Santamaría A. (2018). Redox signaling, neuroinflammation, and neurodegeneration. Antioxid. Redox Signal..

[35] Ko C.H., Shen S.C., Yang L.Y., Lin C.W., Chen Y.C. (2007). Gossypol reduction of tumor growth through ros-dependent mitochondria pathway in human colorectal carcinoma cells. Int. J. Cancer.

[36] Lin C.-W., Yang L.-Y., Shen S.-C., Chen Y.-C. (2007). Igf-i plus e2 induces proliferation via activation of ros-dependent erks and jnks in human breast carcinoma cells. J. Cell. Physiol..

[37] Wu M.S., Chien C.C., Cheng K.T., Subbaraju G.V., Chen Y.C. (2017). Hispolon suppresses lps- or lta-induced inos/no production and apoptosis in bv-2 microglial cells. Am. J. Chin. Med..

[38] Mori G., Brunetti G., Colucci S., Ciccolella F., Coricciati M., Pignataro P., Oranger A., Ballini A., Farronato D., Mastrangelo F. (2007). Alteration of activity and survival of osteoblasts obtained from human periodontitis patients: Role of trail. J. Biol. Regul. Homeost. Agents.

[39] Mori G., Brunetti G., Colucci S., Oranger A., Ciccolella F., Sardone F., Pignataro P., Mori C., Karapanou V., Ballini A. (2009). Osteoblast apoptosis in periodontal disease: Role of tnf-related apoptosis-inducing ligand. Int. J. Immunopathol. Pharmacol..

[40] Kumar A., Yegla B., Foster T.C. (2018). Redox signaling in neurotransmission and cognition during aging. Antioxid. Redox Signal..

[41] Uttara B., Singh A.V., Zamboni P., Mahajan R.T. (2009). Oxidative stress and neurodegenerative diseases: A review of upstream and downstream antioxidant therapeutic options. Curr. Neuropharmacol..

[42] Chuang J.Y., Chang P.C., Shen Y.C., Lin C., Tsai C.F., Chen J.H., Yeh W.L., Wu L.H., Lin H.Y., Liu Y.S. (2014). Regulatory effects of fisetin on microglial activation. Molecules.

[43] Lin C., Lin H.Y., Chen J.H., Tseng W.P., Ko P.Y., Liu Y.S., Yeh W.L., Lu D.Y. (2015). Effects of paeonol on anti-neuroinflammatory responses in microglial cells. Int. J. Mol. Sci..

[44] Bavarsad Shahripour R., Harrigan M.R., Alexandrov A.V. (2014). N-acetylcysteine (nac) in neurological disorders: Mechanisms of action and therapeutic opportunities. Brain Behav..

[45] Khan M., Sekhon B., Jatana M., Giri S., Gilg A.G., Sekhon C., Singh I., Singh A.K. (2004). Administration of n-acetylcysteine after focal cerebral ischemia protects brain and reduces inflammation in a rat model of experimental stroke. J. Neurosci. Res..

[46] Zhang Z., Yan J., Taheri S., Liu K.J., Shi H. (2014). Hypoxia-inducible factor 1 contributes to n-acetylcysteine’s protection in stroke. Free Radic. Biol. Med..

[47] Lu D.Y., Chen H.C., Yang M.S., Hsu Y.M., Lin H.J., Tang C.H., Lee C.H., Lai C.K., Lin C.J., Shyu W.C. (2012). Ceramide and toll-like receptor 4 are mobilized into membrane rafts in response to helicobacter pylori infection in gastric epithelial cells. Infect. Immun..

[48] Lécuyer M.A., Saint-Laurent O., Bourbonnière L., Larouche S., Larochelle C., Michel L., Charabati M., Abadier M., Zandee S., Haghayegh Jahromi N. (2017). Dual role of alcam in neuroinflammation and blood-brain barrier homeostasis. Proc. Natl. Acad. Sci. USA.

[49] Puscas I., Bernard-Patrzynski F., Jutras M., Lécuyer M.A., Bourbonnière L., Prat A., Leclair G., Roullin V.G. (2019). Ivivc assessment of two mouse brain endothelial cell models for drug screening. Pharmaceutics.

[50] Shapira L., Ayalon S., Brenner T. (2002). Effects of porphyromonas gingivalis on the central nervous system: Activation of glial cells and exacerbation of experimental autoimmune encephalomyelitis. J. Periodontol..

[51] Livak K.J., Schmittgen T.D. (2001). Analysis of relative gene expression data using real-time quantitative pcr and the 2(-delta delta c(t)) method. Methods.

[52] Pathirana R.D., O’Brien-Simpson N.M., Visvanathan K., Hamilton J.A., Reynolds E.C. (2007). Flow cytometric analysis of adherence of porphyromonas gingivalis to oral epithelial cells. Infect. Immun..

[53] van Tonder A., Joubert A.M., Cromarty A.D. (2015). Limitations of the 3-(4,5-dimethylthiazol-2-yl)-2,5-diphenyl-2h-tetrazolium bromide (mtt) assay when compared to three commonly used cell enumeration assays. BMC Res. Notes.

[54] Ju T.C., Chen S.D., Liu C.C., Yang D.I. (2005). Protective effects of s-nitrosoglutathione against amyloid beta-peptide neurotoxicity. Free Radic. Biol. Med..

[55] Yang L.Y., Shen S.C., Cheng K.T., Subbaraju G.V., Chien C.C., Chen Y.C. (2014). Hispolon inhibition of inflammatory apoptosis through reduction of inos/no production via ho-1 induction in macrophages. J. Ethnopharmacol..

[56] Pizarro-Cerdá J., Cossart P. (2006). Bacterial adhesion and entry into host cells. Cell.

[57] How K.Y., Song K.P., Chan K.G. (2016). Porphyromonas gingivalis: An overview of periodontopathic pathogen below the gum line. Front. Microbiol..

[58] Stathopoulou P.G., Galicia J.C., Benakanakere M.R., Garcia C.A., Potempa J., Kinane D.F. (2009). Porphyromonas gingivalis induce apoptosis in human gingival epithelial cells through a gingipain-dependent mechanism. BMC Microbiol..

[59] Huang B.R., Tsai C.F., Lin H.Y., Tseng W.P., Huang S.S., Wu C.R., Lin C., Yeh W.L., Lu D.Y. (2013). Interaction of inflammatory and anti-inflammatory responses in microglia by staphylococcus aureus-derived lipoteichoic acid. Toxicol. Appl. Pharmacol..

[60] Lin H.Y., Tang C.H., Chen Y.H., Wei I.H., Chen J.H., Lai C.H., Lu D.Y. (2010). Peptidoglycan enhances proinflammatory cytokine expression through the tlr2 receptor, myd88, phosphatidylinositol 3-kinase/akt and nf-kappab pathways in bv-2 microglia. Int. Immunopharmacol..

[61] Lu D.Y., Tang C.H., Chang C.H., Maa M.C., Fang S.H., Hsu Y.M., Lin Y.H., Lin C.J., Lee W.C., Lin H.J. (2012). Helicobacter pylori attenuates lipopolysaccharide-induced nitric oxide production by murine macrophages. Innate. Immun..

[62] Tsai C.F., Yeh W.L., Huang S.M., Tan T.W., Lu D.Y. (2012). Wogonin induces reactive oxygen species production and cell apoptosis in human glioma cancer cells. Int. J. Mol. Sci..

[63] Grau A.J., Becher H., Ziegler C.M., Lichy C., Buggle F., Kaiser C., Lutz R., Bültmann S., Preusch M., Dörfer C.E. (2004). Periodontal disease as a risk factor for ischemic stroke. Stroke.

[64] Ding Y., Ren J., Yu H., Yu W., Zhou Y. (2018). Porphyromonas gingivalis, a periodontitis causing bacterium, induces memory impairment and age-dependent neuroinflammation in mice. Immun. Ageing I A.

[65] Chen Z., Casiano C.A., Fletcher H.M. (2001). Protease-active extracellular protein preparations from porphyromonas gingivalis w83 induce n-cadherin proteolysis, loss of cell adhesion, and apoptosis in human epithelial cells. J. Periodontol..

[66] Mao S., Park Y., Hasegawa Y., Tribble G.D., James C.E., Handfield M., Stavropoulos M.F., Yilmaz O., Lamont R.J. (2007). Intrinsic apoptotic pathways of gingival epithelial cells modulated by porphyromonas gingivalis. Cell Microbiol..

[67] Desta T., Graves D.T. (2007). Fibroblast apoptosis induced by porphyromonas gingivalis is stimulated by a gingipain and caspase-independent pathway that involves apoptosis-inducing factor. Cell Microbiol..

[68] Sheets S.M., Potempa J., Travis J., Casiano C.A., Fletcher H.M. (2005). Gingipains from porphyromonas gingivalis w83 induce cell adhesion molecule cleavage and apoptosis in endothelial cells. Infect. Immun..

[69] Sheets S.M., Potempa J., Travis J., Fletcher H.M., Casiano C.A. (2006). Gingipains from porphyromonas gingivalis w83 synergistically disrupt endothelial cell adhesion and can induce caspase-independent apoptosis. Infect. Immun..

[70] Maitre Y., Micheneau P., Delpierre A., Mahalli R., Guerin M., Amador G., Denis F. (2020). Did the brain and oral microbiota talk to each other? A review of the literature. J. Clin. Med..

[71] Lamont R.J., Jenkinson H.F. (2000). Subgingival colonization by porphyromonas gingivalis. Oral Microbiol. Immunol..

[72] Boisvert H., Duncan M.J. (2008). Clathrin-dependent entry of a gingipain adhesin peptide and porphyromonas gingivalis into host cells. Cell Microbiol..

[73] Nakagawa I., Amano A., Kuboniwa M., Nakamura T., Kawabata S., Hamada S. (2002). Functional differences among fima variants of porphyromonas gingivalis and their effects on adhesion to and invasion of human epithelial cells. Infect. Immun..

[74] Takahashi Y., Davey M., Yumoto H., Gibson F.C., Genco C.A. (2006). Fimbria-dependent activation of pro-inflammatory molecules in porphyromonas gingivalis infected human aortic endothelial cells. Cell Microbiol..

[75] Mei F., Xie M., Huang X., Long Y., Lu X., Wang X., Chen L. (2020). Porphyromonas gingivalis and its systemic impact: Current status. Pathogens.

[76] Amano A., Sharma A., Sojar H.T., Kuramitsu H.K., Genco R.J. (1994). Effects of temperature stress on expression of fimbriae and superoxide dismutase by porphyromonas gingivalis. Infect. Immun..

[77] Pöllänen M.T., Paino A., Ihalin R. (2013). Environmental stimuli shape biofilm formation and the virulence of periodontal pathogens. Int. J. Mol. Sci..

[78] Doyle K.P., Simon R.P., Stenzel-Poore M.P. (2008). Mechanisms of ischemic brain damage. Neuropharmacology.

[79] Vallabhapurapu S., Karin M. (2009). Regulation and function of nf-kappab transcription factors in the immune system. Annu. Rev. Immunol..

[80] Wang Y.H., Chen K.M., Chiu P.S., Lai S.C., Su H.H., Jan M.S., Lin C.W., Lu D.Y., Fu Y.T., Liao J.M. (2016). Lumbrokinase attenuates myocardial ischemia-reperfusion injury by inhibiting tlr4 signaling. J. Mol. Cell. Cardiol..

[81] Huang J., Upadhyay U.M., Tamargo R.J. (2006). Inflammation in stroke and focal cerebral ischemia. Surg. Neurol..

[82] Wang Q., Tang X.N., Yenari M.A. (2007). The inflammatory response in stroke. J. Neuroimmunol..

[83] Quagliarello V.J., Wispelwey B., Long W.J., Scheld W.M. (1991). Recombinant human interleukin-1 induces meningitis and blood-brain barrier injury in the rat. Characterization and comparison with tumor necrosis factor. J. Clin. Investig..

[84] Grebe A., Hoss F., Latz E. (2018). Nlrp3 inflammasome and the il-1 pathway in atherosclerosis. Circ. Res..

[85] Shafi O. (2020). Switching of vascular cells towards atherogenesis, and other factors contributing to atherosclerosis: A systematic review. Thromb. J..

[86] van Kralingen C., Kho D.T., Costa J., Angel C.E., Graham E.S. (2013). Exposure to inflammatory cytokines il-1β and tnfα induces compromise and death of astrocytes; implications for chronic neuroinflammation. PLoS ONE.

[87] Park E., Na H.S., Song Y.R., Shin S.Y., Kim Y.M., Chung J. (2014). Activation of nlrp3 and aim2 inflammasomes by porphyromonas gingivalis infection. Infect. Immun..

[88] Zhou Q., Desta T., Fenton M., Graves D.T., Amar S. (2005). Cytokine profiling of macrophages exposed to porphyromonas gingivalis, its lipopolysaccharide, or its fima protein. Infect. Immun..

[89] Wang H., Zhou H., Duan X., Jotwani R., Vuddaraju H., Liang S., Scott D.A., Lamont R.J. (2014). Porphyromonas gingivalis induced reactive oxygen species activate jak2 and regulate production of inflammatory cytokines through c-jun. Infect. Immun..

[90] Parajuli B., Sonobe Y., Horiuchi H., Takeuchi H., Mizuno T., Suzumura A. (2013). Oligomeric amyloid β induces il-1β processing via production of ros: Implication in alzheimer’s disease. Cell Death Dis..

[91] Morgan M.J., Liu Z.G. (2011). Crosstalk of reactive oxygen species and nf-κb signaling. Cell Res..

[92] Xu X., Huang X., Zhang L., Huang X., Qin Z., Hua F. (2021). Adiponectin protects obesity-related glomerulopathy by inhibiting ros/nf-κb/nlrp3 inflammation pathway. BMC Nephrol..

[93] Teng J.F., Mei Q.B., Zhou X.G., Tang Y., Xiong R., Qiu W.Q., Pan R., Law B.Y., Wong V.K., Yu C.L. (2020). Polyphyllin vi induces caspase-1-mediated pyroptosis via the induction of ros/nf-κb/nlrp3/gsdmd signal axis in non-small cell lung cancer. Cancers.

[94] Lu D.Y., Chang C.S., Yeh W.L., Tang C.H., Cheung C.W., Leung Y.M., Liu J.F., Wong K.L. (2012). The novel phloroglucinol derivative bfp induces apoptosis of glioma cancer through reactive oxygen species and endoplasmic reticulum stress pathways. Phytomedicine Int. J. Phytother. Phytopharm..

[95] McLoughlin A., Rochfort K.D., McDonnell C.J., Kerrigan S.W., Cummins P.M. (2017). Staphylococcus aureus-mediated blood-brain barrier injury: An in vitro human brain microvascular endothelial cell model. Cell Microbiol..

[96] Rydkina E., Turpin L.C., Sahni A., Sahni S.K. (2012). Regulation of inducible heme oxygenase and cyclooxygenase isozymes in a mouse model of spotted fever group rickettsiosis. Microb. Pathog..

[97] Paterson R.L., Galley H.F., Webster N.R. (2003). The effect of n-acetylcysteine on nuclear factor-kappa b activation, interleukin-6, interleukin-8, and intercellular adhesion molecule-1 expression in patients with sepsis. Crit. Care Med..

[98] Shin J.A., Yoon J.C., Kim M., Park E.M. (2016). Activation of classical estrogen receptor subtypes reduces tight junction disruption of brain endothelial cells under ischemia/reperfusion injury. Free Radic. Biol. Med..

[99] Arcambal A., Taïlé J., Rondeau P., Viranaïcken W., Meilhac O., Gonthier M.P. (2019). Hyperglycemia modulates redox, inflammatory and vasoactive markers through specific signaling pathways in cerebral endothelial cells: Insights on insulin protective action. Free Radic. Biol. Med..

[100] Camós S., Mallolas J. (2010). Experimental models for assaying microvascular endothelial cell pathophysiology in stroke. Molecules.

[101] Bernard-Patrzynski F., Lécuyer M.A., Puscas I., Boukhatem I., Charabati M., Bourbonnière L., Ramassamy C., Leclair G., Prat A., Roullin V.G. (2019). Isolation of endothelial cells, pericytes and astrocytes from mouse brain. PLoS ONE.

