# Supplementary figures and images for: Porphyromonas gingivalis Induces Proinflammatory Cytokine Expression Leading to Apoptotic Death through the Oxidative Stress/NF-κB Pathway in Brain Endothelial Cells
Source: Cells. 2021 Nov 5;10(11):3033. doi: 10.3390/cells10113033 (PMC8616253; doi:10.3390/cells10113033)

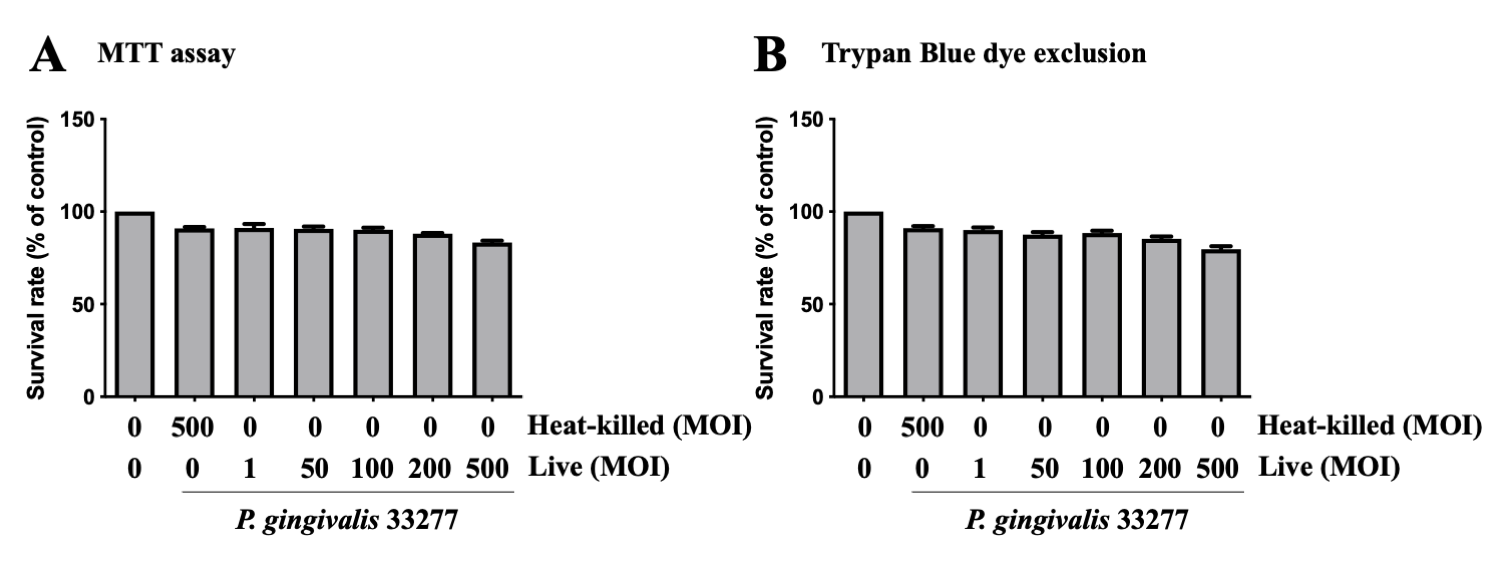

Supplement: Supplementary file 1 [file cells-10-03033-s001.zip › cells-1411579-supplementary.tiff]
